# Effect of Vibration on Alleviating Foot Pressure-Induced Ischemia under Occlusive Compression

**DOI:** 10.1155/2021/6208499

**Published:** 2021-10-25

**Authors:** Weiyan Ren, Mingzheng Zhang, Hongmei Liu, Yih-Kuen Jan, Fang Pu, Yubo Fan

**Affiliations:** ^1^Beijing Key Laboratory of Rehabilitation Technical Aids for Old-Age Disability, Key Laboratory of Human Motion Analysis and Rehabilitation Technology of the Ministry of Civil Affairs, National Research Center for Rehabilitation Technical Aids, Beijing, China; ^2^Affiliated Hospital of National Research Center for Rehabilitation Technical Aids, Beijing, China; ^3^Beijing Advanced Innovation Centre for Biomedical Engineering, School of Biological Science and Medical Engineering, Beihang University, Beijing, China; ^4^Rehabilitation Engineering Laboratory, Department of Kinesiology and Community Health, University of Illinois at Urbana-Champaign, Champaign, IL, USA; ^5^School of Engineering Medicine, Beihang University, Beijing, China

## Abstract

**Objectives:**

Foot ulcers often occur in people with diabetes because of pressure-induced tissue ischemia. Vibration has been reported to be helpful in alleviating mechanical damage and promoting wound healing. The objective of this study is to explore whether vibration can relieve reactive hyperemia in foot tissue under occlusive compression.

**Methods:**

Thirteen healthy adults participated in the study. Each foot was placed under occlusive compression without or with vibration intervention, which was randomly assigned every other day. The dorsal foot skin blood flow (SBF) was measured pre- and postintervention for each subject in each test. Temporal variations and spectral features of SBF were recorded for comparison.

**Results:**

The results showed that subjects displayed an obvious reactive hyperemia in the foot tissue after pressure occlusion, whereas they displayed a more regular SBF when vibration was applied along with occlusive compression. Moreover, the amplitude of metabolic, neurogenic, and myogenic pathways for SBF was significantly reduced during the hyperemia process when vibration was applied.

**Conclusions:**

This study demonstrated that vibration can effectively reduce the level of hyperemia in foot tissue under occlusive compression and also induce less protective physiological regulatory activities. This is helpful for protecting foot tissue from pressure-induced ischemic injury and foot ulcers.

## 1. Introduction

Diabetic foot ulcers are one of the most serious complications for diabetics, with an incidence rate of approximately 15% in the diabetic population [1]. Tissue ischemia is one of the main causes of pressure ulcers [2]. When the external pressure applied to foot tissue exceeds the capillary blood pressure, the resulting occlusion of blood and lymph vessels causes changes in the metabolism and can lead to the accumulation of waste products [1, 3, 4]. However, when the pressure is removed, the increased blood flow reduces the intensity of the ischemia [3, 5, 6]. Particularly in diabetics with impaired microcirculation, the increased plantar skin blood flow during reactive hyperemia may not be sufficient to compensate for the hypoxia-ischemia and accumulation of metabolic waste in the tissues, which can increase the risk of developing infections and ulcerations [7–9]. Thus, an effective method to alleviate ischemic damage and improve tissue viability under prolonged pressure stimuli would be of considerable benefit to diabetics.

Previous studies have demonstrated the effectiveness of vibration for alleviating mechanical and oxidative damage, promoting angiogenesis and wound healing, and increasing microcirculation. Wong et al. applied prolonged compression (100 mmHg, 6 h) to the biceps femoris of mice and investigated differences in tissue reactions with and without the use of whole body vibration intervention (35 Hz, acceleration 0.25 g) [10]. Wong found that the mechanical and oxidative damage was significantly reduced following vibration intervention, indicating the effectiveness of vibration for maintaining antioxidative defense and relieving ischemic injury during persistent mechanical stress. Weinheimer-Haus et al. reported that the whole body low-intensity vibration could promote angiogenesis and granulation and facilitate the expression of growth factors and chemokines in wounded tissue of diabetic mice, which can accelerate wound healing [11]. Using rat models to investigate the biological effect of vibration intervention, Sari et al. found that whole body vibration could inhibit hypoxia and matrix metalloproteinase activity and effectively attenuate the deterioration of deep tissue injury [12]. However, these studies demonstrated the effectiveness of vibration for alleviating mechanical damage in rats, which may not be truly representative of the reaction in humans. Although Wilson et al. and Midori et al. reported that local vibration on lower extremity could facilitate the healing rate of ulceration in patients with venous leg ulcers and stage I pressure ulcers [13, 14], it is not yet known whether vibration could relieve ischemia caused by pressure stimulus. Nakagami et al. pointed out that promoting blood flow during the compression of tissues may alleviate the degree of ischemia in occluded tissues [15]. Also, Maloney-Hinds et al. found that local vibration on the arm could increase the skin blood flow of intervention limb [16]. Thus, we speculated that vibration could also relieve the ischemia in the lower extremities caused by pressure stimulus. Moreover, its impact on the postocclusive hyperemia and regulatory mechanisms has not yet been investigated.

Previous studies considered the hyperemia response after the release of compression as a potential method for evaluating the degree of ischemia in occluded tissues [15, 17]. Normally, the level of postocclusive hyperemia is correlated with the degree of tissue ischemia [15, 18, 19]. Skin blood flow (SBF) has also been widely used to assess vasodilation of skin vessels and the degree of skin hyperemia [20, 21]. Wavelet analysis of SBF oscillations can display the physiological mechanisms of blood flow regulation. For instance, SBF frequency bands ranging between 0.0095–0.02 Hz, 0.02–0.05 Hz, and 0.05–0.15 Hz are associated with endothelial-dependent metabolic activity, neurogenic controls of the vessel wall, and myogenic activity of vascular smooth muscles in the local tissue, respectively [21–24]. Thus, these characteristics which are embedded in the SBF oscillations can be used to investigate the physiological regulatory mechanisms of local reactive hyperemia under vibration intervention.

The objective of this study is to investigate the effects of vibration on postocclusive hyperemic foot tissue. We hypothesized that vibration intervention could reduce the level of reactive hyperemia response in foot tissue when placed under pressure occlusion.

## 2. Materials and Methods

### 2.1. Participants

Thirteen healthy adults (5 males and 8 females) participated in this study. The inclusion criteria were as follows: (1) had no symptoms such as redness, callus, inflammation, or wounds on the skin of the foot or legs and (2) had no diseases such as hypertension, peripheral neuropathy, vascular diseases, heart diseases, systematic inflammation, and malignant tumors. This study was conducted in accordance with clinical protocols approved by the institutional review board of Affiliated Hospital of National Research Center for Rehabilitation Technical Aids and conducted in accordance with the Declaration of Helsinki. All subjects gave informed written consent prior to participation.

### 2.2. Test Equipment

**Figure 1:**
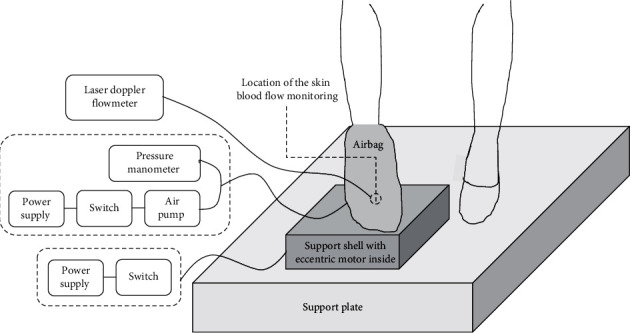
Test setup for applying pressure and vibration to the whole foot and measuring skin blood flow of the dorsum of foot.

Custom-designed devices were developed to apply whole foot compression and whole foot vibration in this study. Compression was applied using an air operated pressure device consisting of an airbag, pressure manometer, air pump, power supply, and a switch. When placed on the foot, the pressure was increased to 150 mmHg (calibrated by using a mercury gauge) and then maintained for 15 min at this level in order to provide compression stimuli without discomfort and injury [25, 26]. The vibration device consisted of an eccentric motor, controller, support shell, power supply, and a switch. The test setup is shown in Figure 1. As vibration with a frequency of 50 Hz and an amplitude of 2 mm was proven to effectively improve microcirculation and wound healing [27], these parameters were chosen to verify the effect of vibration on alleviating pressure-induced ischemia. The compression and continuous vibration were applied to the whole right foot of each subject. The SBF in the dorsum of the right foot was measured by using a laser Doppler flowmeter (PeriFlux 5001, Probe 457, Perimed, Stockholm, Sweden) at a sampling frequency of 32 Hz [28].

### 2.3. Test Protocol

Two tests were developed for this study: (1) No Vibration test, where only a whole foot occlusive compression stimulus (150 mmHg, 15 min) was applied to the subjects' right foot, and (2) Vibration test, where vibration intervention (50 Hz, 2 mm, 15 min) was applied to the whole foot when under occlusive compression (150 mmHg, 15 min). Each subject received both tests in a random order over two days, one test each day. Before each test, the subject was asked to rest in a room at a temperature of 24 ± 2°C for 30 min.

**Figure 2:**
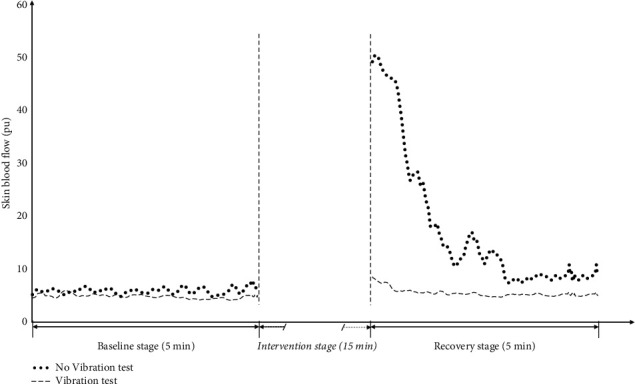
Typical example of the variation in skin blood flow during the baseline and the recovery stage with and without vibration in two tests.

There are three basic stages to each test: (1) *baseline stage* is the first 5 min of the test right immediately after the resting period where the compression and vibration devices are attached to the patient but are not activated; (2) *intervention stage* occurs after the baseline stage, during which a whole foot occlusive compression stimulus (150 mmHg, 15 min) was applied to each subject, with the vibration intervention in the Vibration test or without in the No Vibration test [10]; and (3) *recovery stage* is a final 5 min period when the compression and vibration stimuli are deactivated and the subject is allowed to rest. The dorsal SBF of each subject's right foot was measured continuously throughout the entire test when in a seated position using a flowmeter sensor placed on the midshaft between the 2^nd^ and 3^rd^ metatarsal [29–31].  Figure 2 shows a typical example of the variation in SBF with and without vibration during the baseline and the recovery stage, in which the SBF with less physiological significance in the intervention stage was removed.

### 2.4. Data Analysis

The relatively stable readings during the baseline stage permitted the SBF to be characterized by its mean value (see Figure 2). For the recovery stage, the mean SBF was recorded every minute (total 5 min). The percentage change in SBF during the recovery stage relative to the baseline stage was calculated for further analysis.

Wavelet analyses of SBF oscillations during baseline and recovery stages were evaluated to gain insight into changes in the underlying SBF regulatory activities pre- and postintervention. The SBF oscillations are regulated by five physiological mechanisms with frequencies ranging from 0.0095 to 2 Hz, relating to regulatory components of metabolic, neurogenic, myogenic, respiratory, and cardiac origins, respectively [22, 23]. However, this study focused on the three major local physiological regulatory mechanisms: endothelial-dependent metabolic activities (0.0095–0.02 Hz), neurogenic activities (0.02–0.05 Hz), and myogenic activities (0.05–0.15 Hz).

**Figure 3:**
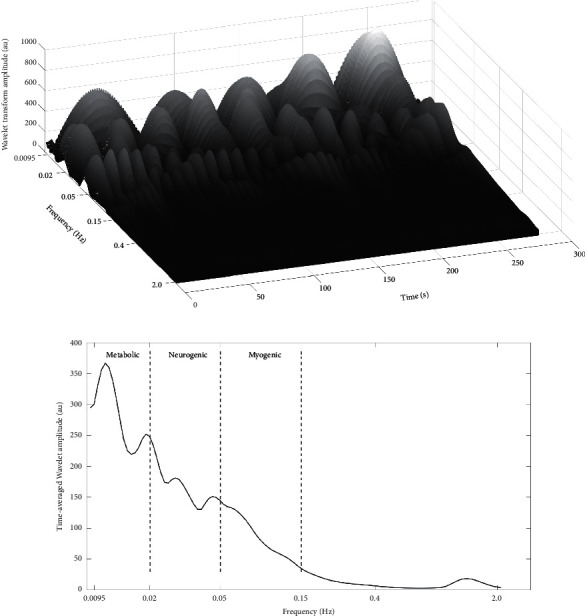
An example of the wavelet transforms (a) and time-averaged wavelet amplitudes (b) of the skin blood flow signal.

The continuous wavelet transform of a signal *f*(*u*) of skin blood flow was defined as

(1) $$\begin{array}{c} f\left(s,t\right)=\int_{-\infty }^{\infty }\psi_{s,t}\left(u\right)f\left(u\right)du, \end{array}$$

where *ψ*_*s*,*t*_(*u*) is a wavelet function and is defined as

(2) $$\begin{array}{c} \psi_{s,t}\left(u\right)=\frac{1}{\sqrt{s}}\psi \left(\frac{u-t}{s}\right), \end{array}$$

where *ψ* is the mother wavelet function, *t* is the time factor, and *s* is the scaling factor. A Morlet wavelet model was used to achieve a continuous wavelet transform.  Figure 3 shows an example of the wavelet transform of the skin blood flow signal. To overcome individual variations between subjects, the amplitude of SBF oscillations during the recovery stage was normalized to that of the baseline stage. The normalized wavelet amplitudes were compared between the two tests (Vibration and No Vibration) to explore the effect of vibration intervention on these physiological regulatory activities.

### 2.5. Statistical Analysis

The Wilcoxon matched-pair signed-rank test was used to assess differences in SBF response pre- and postintervention. The Mann–Whitney *U* test was used to analyze differences in percentage change of SBF and its underlying regulatory activities (characterized by the normalized wavelet amplitudes within specific frequency bands) between two tests. The level of significance was set at 0.05. All statistical analyses were performed in SPSS (version 20; IBM, Armonk, NY, USA).

## 3. Results

All recruited subjects participated in and completed the experiments. All subjects' details are shown in Table 1.

**Figure 4:**
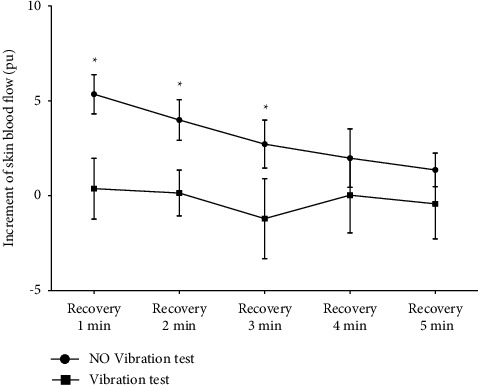
Increment of SBF during the recovery stage between the No Vibration test and the Vibration test. ∗indicates a significantly greater increment of SBF for the No Vibration test compared to the Vibration test in the corresponding period. ^*∗*^*p* < 0.05.

The increments of SBF for both tests are illustrated in Figure 4. The results showed that the increment of SBF during the first 3 min of the recovery stage in the No Vibration test was significantly greater than that in the Vibration test, which implied a distinct hyperemia was observed in the No Vibration test but not in the Vibration test.

**Figure 5:**
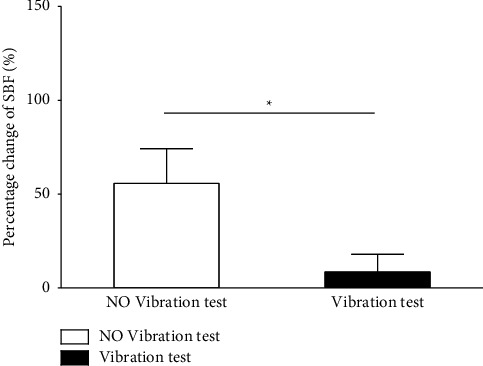
Percentage change in foot SBF between the baseline and recovery stages between the No Vibration test and the Vibration test. ∗indicates a significantly greater percentage change in SBF for the No Vibration test compared to the Vibration test. ^*∗*^*p* < 0.05.

From Figure 5, it can be seen that there was a significantly greater percentage change in foot SBF between the baseline and recovery stages for the No Vibration test than the Vibration test. This indicated that applying vibration while the tissue was compressed could help to reduce the severity of pressure-induced reactive hyperemia.

**Figure 6:**
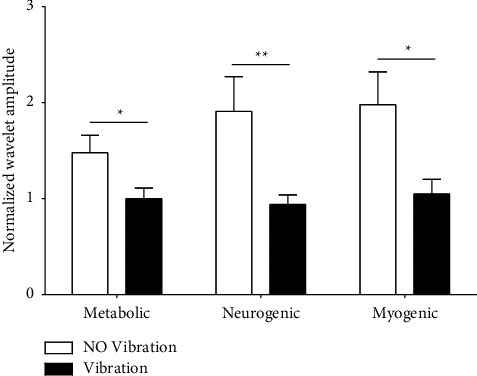
Normalized wavelet amplitudes of foot SBF oscillations during the recovery stage in the No Vibration test and the Vibration test. ∗indicates a significantly higher normalized wavelet amplitude for the No Vibration test compared to the Vibration test. ^*∗*^*p* < 0.05 and ^*∗*^^*∗*^*p* < 0.01.

The normalized wavelet amplitudes of SBF oscillations associated with metabolic, neurogenic, and myogenic regulation pathways are shown in Figure 6. The results show that the amplitudes for all three physiological pathways were significantly higher in the No Vibration test than in the Vibration test (*p* < 0.05).

## 4. Discussion

This study investigated the effects of vibration intervention on the hyperemia response and its regulatory mechanisms in foot tissue under occlusive compression. The results showed that vibration could reduce the level of reactive hyperemia induced by persistent compression, which may be attributed to the changes in physiological regulatory activities.

When excessive pressure is applied to the foot, microvessels in the skin become blocked, leading to the accumulation of xanthine oxidase. The oxygen-starved hypoxic tissue needs a massive flow of blood to provide nutrition and remove metabolic waste. Once the occlusion is removed, a reactive hyperemia occurs to provide oxygen to ischemic tissue and remove waste [3, 4]. The healthy subjects in this study can be assumed to have a normal functioning nervous system and endothelial regulation, and thus a distinct reactive hyperemia was observed when the feet were occluded, which compensates for the compression-induced ischemia (Figures 4 and 5; No Vibration test). The blood flow responses in the Vibration test did not display the same hyperemic reaction, which may be attributed to the protective effect of vibration intervention on ischemic tissue during compression.

Pressure-induced vasodilation of local microvascular (reflected as postocclusive reactive hyperemia) is known to be regulated by metabolic, neurogenic, and myogenic pathways [22, 23]. Metabolic regulation is mainly related to endothelial activities [32] and will regulate arterioles when changes occur to the metabolite, pressure and/or flow of the vessels [33]. Sympathetic nerves can regulate blood flow by releasing neurotransmitters which act on endothelial cells and vascular smooth muscle [34, 35], and the change in metabolic demand will modulate neurogenic vasomotor tone to control microvascular perfusion [33]. Myogenic activities control the rhythmic constriction and dilation of vasomotion by vascular smooth muscles and are a significant factor in reactive hyperemia [36]. In the presence of hyperemia, myogenic regulation is activated to modulate the diameter of vessels and blood flow resistance and to prevent excessive pressure in the capillary network [33, 37, 38]. Prolonged epidermal loading, through compression in this study, can disturb the blood flow and related regulations in the microvascular network, leading to changes in metabolic demands, nervous activities, and vasomotion [39, 40]. A reduction in the oxygen supply to the occluded tissues is the principal cause of reactive hyperemia. In order to meet the increased metabolic demand in occluded tissues, the three pathways (metabolic, neurogenic, and myogenic) cooperated to regulate vasodilation and control blood flow [33]. Previous studies reported that vibration could reduce pressure-induced damage in compressed tissue by maintaining enzymatic oxidation defenses and inducing vasodilatation [2, 4, 41]. Thus, compared to the No Vibration test, less blood flow supply and related physiological regulations are needed to compensate for the pressure-induced ischemia and disturbed circulation after the release of compression in the Vibration test. In this study, the postocclusive reactive hyperemia and related wavelet amplitudes (metabolic, neurogenic, and myogenic origins) were all significantly reduced when subjects received compression stimulation with vibration, indicating vibration could help to weaken such compensatory responses and alleviate pressure-induced ischemia.

Postocclusive reactive hyperemia is a common method to assess peripheral vascular function in people with diabetes [42, 43] and estimate therapeutic effect [44]. By drawing on this inflation-compression model, this experiment applied compression with 150 mmHg for 15 min to subjects' whole foot and measured microvascular responses under vibration/nonvibration to explore whether vibration could alleviate pressure-induced ischemia. In this experiment, the pressure applied to the skin needs to be greater than the capillary pressure in order to occlude the microvasculature [45–47], meaning a pressure of at least 120 mmHg is required for blocking blood flow in healthy seated adults [2, 48]. Studies have shown that compression with 150 mmHg does not induce discomfort or distraction [25] or lead to tissue damage [49]. This value is also less than the ankle systolic blood pressure (170 mmHg) and the safety threshold of in-shoe pressure [49–51]. Winsor applied 15 min cuff occlusion at the ankle to determine arterial insufficiency [44]. Pu et al. used the cumulative pressure of 15 min natural walking in people with diabetes as the stimulus dose to investigate plantar microvascular responses of diabetic foot [26]. Moreover, the accumulative compression dose of 150 mmHg for 15 min would not cause microcirculation pathological damage [52]. Therefore, this experiment applied compression at 150 mmHg for 15 min to induce postocclusive hyperemia.

Vibration with a frequency of 50 Hz was reported to improve microcirculation and wound healing [16, 53]. The depth of nutritive capillaries in the plantar dermal layer is less than 3 mm [54], and 50 Hz vibration with an amplitude of 2 mm was proven to effectively increase plantar skin blood flow in people with diabetes [27]. Thus, a continuous vibration with a frequency of 50 Hz and an amplitude of 2 mm was selected to provide protective intervention for the whole foot to verify its effect on alleviating pressure-induced ischemia. The impaired endothelial function and disrupted nerve conduction pathway in diabetics often affect the protective microvascular responses to mechanical stimulus [20]. Thus, it is necessary to relieve pressure-induced ischemia to avoid ischemic injury and foot ulcers in people with diabetes. This study demonstrated that vibration could, to some extent, alleviate the degree of ischemia in foot tissue caused by compression stimuli. This suggests that vibration could be effectively used to protect foot tissue from pressure-induced ischemia and ulceration.

There are some limitations to this study that should be noted. First, in this preliminary study, only healthy subjects were tested. Future work may consider diabetic subjects to further verify its clinical effectiveness. Second, this study examined the skin of the dorsal foot where there would be little intersubject variation in tissue hardness because the hardness of skin tissue may affect the microvascular response. This may be investigated in future studies by recruiting diabetic subjects with similar plantar hardness. Third, the method of pressure stimulation needs to be improved and be closer to daily activities in the future study.

## 5. Conclusion

In this study, occlusive compression and vibration were applied to the foot to examine the effectiveness of vibration treatment on alleviating reactive hyperemia responses. The results showed that vibration could effectively reduce the level of hyperemia after prolonged whole foot occlusion, which was attributed to the weakened protective regulatory activities of the metabolic, neurogenic, and myogenic pathways.

## Figures and Tables

**Table 1:** Demographic and physiological information of subjects.

Variable	Value (mean ± SD)
Gender (male/female)	5/8
Age (years)	23.00 ± 0.58
BMI (kg/m^2^)	20.61 ± 2.91
SBP (mmHg)	118.62 ± 9.50
DBP (mmHg)	68.54 ± 7.62
Heart rate (bpm)	74.77 ± 10.73
Ankle-brachial index	1.03 ± 0.07

BMI, body mass index; SBP, systolic blood pressure; DBP, diastolic blood pressure.

## Data Availability

All data are available upon request to the corresponding author Fang Pu via e-mail (pufangbme@buaa.edu.cn).
